# Retraction and replacement of ‘Genomic context-dependent histone H3K36 methylation by three Drosophila methyltransferases and implications for dedicated chromatin readers’

**DOI:** 10.1093/nar/gkaf243

**Published:** 2025-04-01

This is a retraction and replacement of: Muhunden Jayakrishnan, Magdalena Havlová, Václav Veverka, Catherine Regnard, and Peter B. Becker, Genomic context-dependent histone H3K36 methylation by three *Drosophila* methyltransferases and implications for dedicated chromatin readers, *Nucleic Acids Research*, Volume 52, Issue 13, 22 July 2024, Pages 7627–7649, https://doi.org/10.1093/nar/gkae449

In December 2024, the authors requested retraction and replacement of their article, after discovering a software bug in the spike-in ChIP-seq processing pipeline. As a result, while all ChIP-seq profiles analyzed in the manuscript were normalized to the total number of reads and to the corresponding input, they were not additionally scaled to the *D.virilis* spike-in reads as reported originally.

The authors reprocessed data with a corrected pipeline (deleting the ‘-single’ parameter in HOMER makeTagDirectory), which verified that in general, spike-in normalization does not lead to changes in large-scale patterns but enhances the effect size of the global decrease in histone modifications upon RNA interference of histone methyltransferase (HMT) expression. This effect was noticeably pronounced for K36me2, the most widely distributed mark out of all factors interrogated in this study. With the more sensitive quantification, the authors observed additional subtle contributions of each HMT in regions that were not previously associated with the corresponding enzyme, necessitating a reanalysis, as well as a slight revision of their model. This affected Figures 3 (B-D), 4 (A, B, D) and 6 (A-C), along with Supplementary Figures S4, S5, S10 (B), S12 and S13 (B-F). While the majority of the conclusions described in the original manuscript remain valid following reanalysis, the authors updated the paper, inserting corrected figures after spike-in normalization and accordingly revising descriptions and interpretation of the data, and in the ‘Materials and Methods’ section, both providing updated code and explaining the revised procedure for normalization and scaling.

The new version was peer-reviewed and accepted by the editors and has been republished at https://doi.org/10.1093/nar/gkaf202.

The Editors commend the Authors for being forthcoming and disclosing this issue and for reprocessing their data.

